# Health-related quality of life and its association with medication adherence in active pulmonary tuberculosis– a systematic review of global literature with focus on South Africa

**DOI:** 10.1186/s12955-016-0442-6

**Published:** 2016-03-11

**Authors:** Tanja Kastien-Hilka, Ahmed Abulfathi, Bernd Rosenkranz, Bryan Bennett, Matthias Schwenkglenks, Edina Sinanovic

**Affiliations:** Swiss Tropical and Public Health Institute (Swiss TPH), Socinstr. 57, 4051 Basel, Switzerland; University of Basel, Basel, Switzerland; Health Economics Unit, School of Public Health and Family Medicine, Faculty of Health Sciences, University of Cape Town, Cape Town, South Africa; Division of Clinical Pharmacology, Faculty of Medicine and Health Sciences, Stellenbosch University, Stellenbosch, South Africa; Endpoint Development and Outcomes Assessment, Adelphi Values, Bollington, UK; Institute of Pharmaceutical Medicine, University of Basel, Basel, Switzerland; Epidemiology, Biostatistics and Prevention Institute, University of Zürich, Zürich, Switzerland

**Keywords:** Health-related quality of life, Medication adherence, Tuberculosis, Patient-reported outcomes, South Africa

## Abstract

**Introduction:**

Tuberculosis (TB) is a leading cause of morbidity and mortality in South Africa. Clinical parameters are important objective outcomes in TB; however they often are not directly correlated with subjective well-being of the patient, but can be assessed using patient-reported outcome (PRO) measures. Health-related quality of life (HRQOL) is a specific PRO generally multi-dimensional in nature and includes physical, mental and social health domains. The inclusion of HRQOL PROs in trials and clinical practice can provide additional information beyondclinical and microbiological parameters. Furthermore, HRQOL may be associated with medication adherence. This review focuses on patient-reported HRQOL and its association with medication adherence in TB patients in South Africa.

**Methods:**

A comprehensive search strategy was developed focusing on the impact of TB on patient-reported HRQOL,the existence of a conceptual framework of TB-specific HRQOL, determinants of medication adherence and the association of HRQOL with medication adherence. Data were extracted from all identified articles and additionaldata extraction was performed by two independent reviewers with special focus on longitudinal studies in order to understand changes of HRQOL and adherence over time. Research gaps were identified with regard to patient-reported HRQOL and medication adherence.

**Results:**

A total of 66 articles met the eligibility criteria. Ten HRQOL studies and one adherence study used a longitudinal design, none of these in South Africa. A variety of different generic and disease-specific HRQOL measures were identified in the articles. In South Africa four HRQOL and five adherence studies (non-longitudinal) were published. Similar factors (socio-demographic, socio-economic, disease-related, therapy-related and psycho-social aspects) affect HRQOL and adherence. Although standard TB treatment improved all health domains, psychological well-being and social functioning remained impaired in microbiologically cured patients after treatment.

**Conclusion:**

While evidence of TB impact on HRQOL and medication adherence and their association exists, it is verylimited for the South African situation. No valid and reliable TB-specific HRQOL measures were identified in this systematicreview. An assessment of HRQOL in TB patients in South Africa is required as this may assist with improving current disease management programmes, medication adherence and national treatment guidelines.

**Electronic supplementary material:**

The online version of this article (doi:10.1186/s12955-016-0442-6) contains supplementary material, which is available to authorized users.

## Background

The global burden of tuberculosis (TB) is still a major public health concern although the United Nation’s Millenium Development Goals (MDGs) target to reverse TB incidence by 2015 has been achieved. Around 9.6 million new TB cases and 1.5 million TB deaths were estimated to occur in 2014 worldwide [1]. Twenty-two high-burden countries defined by the World Health Organization (WHO) account for 80 % of all TB cases. Despite the availability and affordability of effective TB medication South Africa has the highest prevalence and incidence rates (696 and 834 cases per 100,000 population) among these countries [1] TB is South Africa’s leading cause of mortality (134 cases per 100,000 population). TB is known to impact health-related quality of life (HRQOL) [2–4]. Effective treatment, relapse and the emergence of multi-drug resistant TB (MDR-TB) are closely related to TB treatment adherence and consequent HRQOL [5]. The assessment of an association between both, HRQOL and medication adherence in TB, would provide valuable information on treatment effectiveness, optimal disease management and health policy making.

Patient-reported outcomes (PRO’s) provide unique evidence of different aspects of the experience of living with a disease or condition and how important these aspects are to patients. In this sense, they go beyond clinical parameters and respect the integrated nature of health, ideally encompassing physical, mental and social well-being. The measurement of HRQOL using PRO’s allows for a multidimensional understanding of health, an evaluation of disease and treatment impact on the health condition and the patients’ daily life. It is strongly related to the World Health Organization’s (WHO’s) definition of health as “*a state of complete physical, mental and social well-being and not merely the absence of disease or infirmity”* [6]. A comprehensive knowledge of HRQOL in TB patients can allow for identification of treatment gaps. Addressing these gaps will lead to improvement of health care services and disease prevention strategies, and support health policy making. A number of countries such as the UK, Germany and Australia rely on PRO data about medical interventions for pricing, reimbursement and health policy decision-making about medical interventions. South Africa is becoming increasingly aware of the importance of such outcome evaluations and has released its first guidance on pharmacoeconomic submissions in February 2013 [7]. The aim of this systematic review was to understand HRQOL and medication adherence during TB treatment and how both concepts are associated based on international literature. Longitudinal studies were of particular interest to understand changes in HRQOL and adherence during the course of TB treatment. The focus of this research lies on active pulmonary TB and not on latent TB (LTBI), MDR-TB, XDR-TB, TB in children or TB with HIV co-infection, as these types of TB show different HRQOL outcomes [8]. As South Africa suffers from a major TB burden, we put a specific focus on HRQOL and adherence to TB treatment in the South African health setting.

## Review

### Methods

#### Search strategy for identification and selection of relevant studies

A systematic literature search has been performed in PubMed, EMBASE and PsychINFO, with the last search conducted on 22 February 2015. Search terms applied included *tuberculosis, health related quality of life, HRQOL, quality of life, South Africa, patient-reported outcomes, outcome assessment, life quality, well-being, adherence, non-adherence and compliance*; different combinations were used (Table 1 in the Additional file 1). Each search term combination resulted in different initial hits which were screened by title and abstract. Articles were excluded if they were not related to the pre-defined search terms or were published in a language other than English. Duplicates were removed. The full texts of all remaining articles were reviewed. References cited by the identified publications were additionally scanned for relevant studies. Data on TB and adherence was additionally taken from the WHO and the Department of Health of the Republic of South Africa. Data from articles with a longitudinal study design were separately extracted and included when psychometric validity and reliability were reported for HRQOL or adherence measures, when changes in HRQOL during TB treatment including at least baseline and end of treatment were reported, and when the study population consisted of new TB cases treated as outpatients.

#### Data quality and data extraction

Data from all identified articles was extracted and structured according to physical, mental and social health aspects of HRQOL; data on medication adherence was summarized. Research on HRQOL and medication adherence in South Africa was described separately. In addition, studies with a longitudinal design were subject to a separate; subsequent data extraction performed by two independent reviewers. This additional extraction covered the PICOS elements (P = population, I = intervention, C = comparator or control, O = outcome, S = study design), with additional items covering major research topic, study objective, study setting, sample size, PRO measure applied and measurement time points. Prior to data extraction, all longitudinal studies underwent quality assessment. As longitudinal studies were observational in nature, quality of reporting was evaluated using the Strengthening the Reporting of Observational Studies in Epidemiology (STROBE) statement [2]. A STROBE quality checklist with 8 items was applied, with scores ranging from 0 (no quality) to 16 (best quality) [2].

### Results

**Fig. 1:**
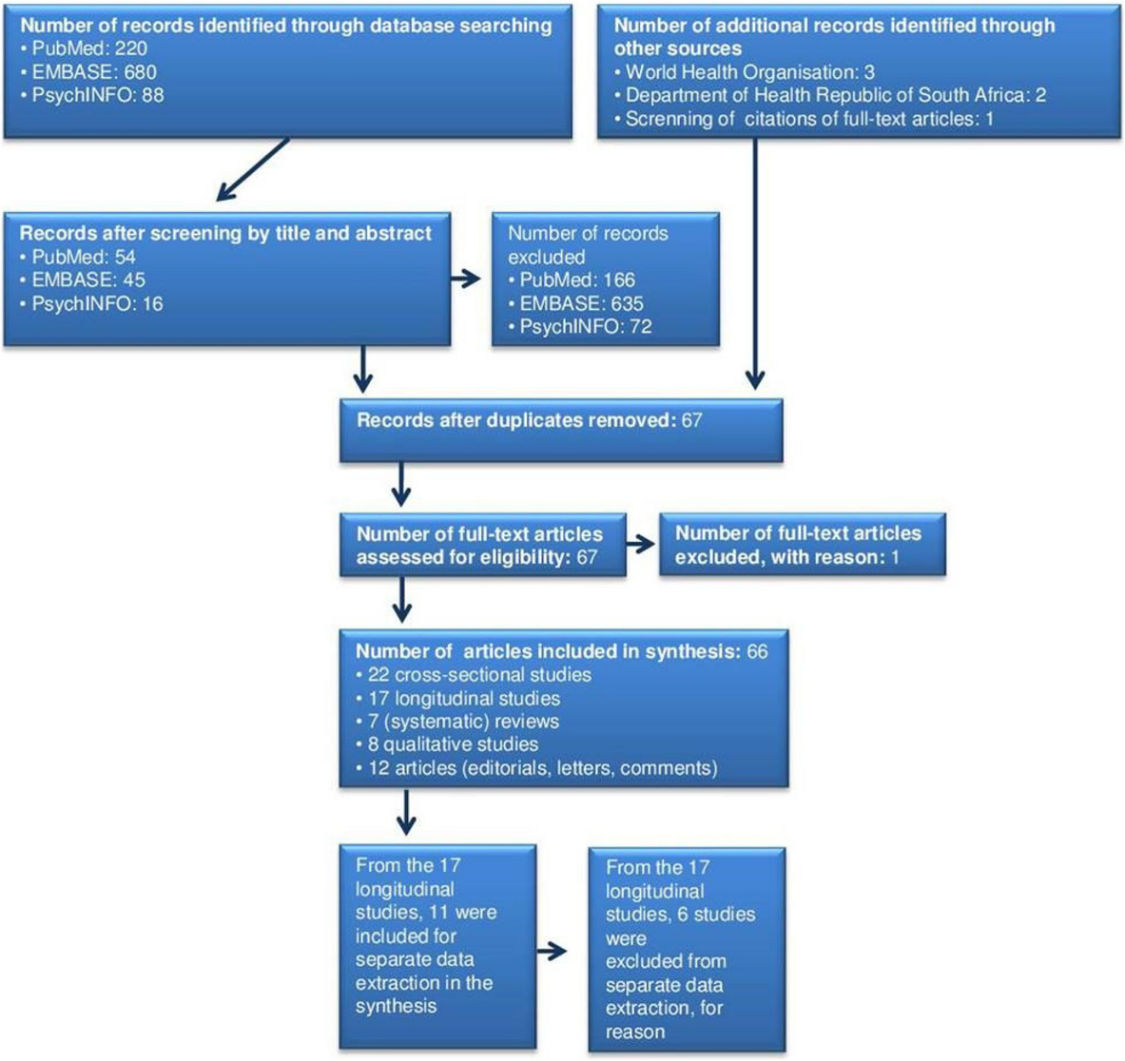
Flow diagram of literature search in the databases PubMed, EMBASE and PsychINFO; additional articles were obtained from World Health Organization and Department of Health Republic of South Africa

Our literature search in PubMed, EMBASE and PsychINFO yielded 988 initial hits. After screening by title and abstract and after removal of duplicates, 61 articles remained. An addition of three WHO reports, two guidelines from the South Africa’s Department of Health and one article identified by hand search of citations resulted in 67 eligible articles for this systematic review. One article was excluded, yielding 66 eligible full-text articles (Fig. 1). A detailed description of the literature search is available in the Additional file 1: Figure 1-4. The 66 articles comprised 22 cross-sectional studies, 17 longitudinal studies, 7 (systematic) reviews, 8 qualitative studies and 12 articles including editorials, comments and letters (Table 2 in the Additional file 1). Nine studies were performed in South Africa (four HRQOL and five medication adherence studies; Table 4 in the Additional file 1). All final 66 articles underwent extraction of information about HRQOL and adherence in TB. The 17 identified longitudinal studies were potentially eligible for separate data extraction; 11 of them actually met the eligibility criteria for separate data extraction, while 6 studies were excluded as eligibility criteria were not met (Table 3 in the Additional file 1). Application of the STROBE quality of reporting checklist to the 11 longitudinal studies resulted in a median score of 7 for HRQOL studies, with scores ranging from 5 to 11 out of 16 (the greater the score the higher the quality of reporting). The adherence study had a score of 10 (Table 3 in the Additional file 1).

#### TB impact on HRQOL

The systematic review found that TB has a negative impact on patients’ HRQOL and overall wellbeing [3, 9–13]. Factors associated with HRQOL in TB included socio-demographic (age, gender) and socio-economic (income, education, housing, social security) factors, disease-related (symptoms) and therapy-related (side effects, adverse events) factors and psycho-social aspects (isolation and stigmatization, psycho-social burden) [2, 4, 9, 10, 13–30]. There was some evidence to suggest that amongst TB patients, psycho-social burden may have a greater impact than clinical symptoms [3, 13]. The results of this literature review identified that TB treatment resulted in a significant improvement in HRQOL, especially in physical and psychological dimensions [2, 4, 10, 17–19, 22, 23, 27, 29]. The improvement in HRQOL was greatest during the first 2–3 months (intensive treatment phase) [2, 23]. The results also revealed that although a patient was defined as microbiologically cured following successful treatment, morbidity still existed due to anatomic and functional changes of the lung at treatment completion [17, 31–33]. Specifically, a state of chronic morbidity continued which resulted in health quality loss that differed for developing countries with regard to their socio-demographic and -economic situation [34–36]. It was also noted that the assessment of HRQOL has currently not been integrated into the WHO guidelines for TB treatment, national guidelines or TB control programmes [37]. An important consideration from the results of the review was that patient-reported HRQOL outcomes may differ after the end of treatment depending on the HRQOL measures applied as different measures may measure different concepts [4, 10, 19, 38–40].

#### TB impact on physical health

TB impacts physical health, resulting in impaired physical functioning, development of fatigue, adverse events of treatment and increased use of health care services. Major physical impairment was reported through somatic symptoms and other TB related physiological outcomes [3, 4, 13, 18]. The impairment in HRQOL was worse in patients with HIV co-infection [15]. Impaired physical functioning was closely related to the development of fatigue [3, 13]. The literature revealed that fatigue was triggered through sleep disturbances, coughing and malnutrition and TB medication [13]. Drug-based treatment of TB impacted HRQOL in two ways. Whilst the TB treatment resulted in a significant improvement in HRQOL [2, 4, 10, 17–19, 22, 29], the drugs taken often resulted in adverse drug reactions which negatively affected HRQOL [13]. The greatest physical impact on HRQOL was caused by the large quantity of pills and the treatment duration, and a reduction in both was expected to improve HRQOL [13]. Health care services may have a negative effect on HRQOL through the relationship between healthcare worker and patient, especially due to a lack of knowledge and misunderstanding about TB. This relationship was not only important for the degree of compliance with the current TB treatment guidelines, but also comprised emotional support and disease-related information through nurses [16, 41].

#### TB impact on mental health

The impact of TB on mental health related to psychological, emotional and spiritual wellbeing and patients perceptionof their health. Psychological distress was commonly reported among TB patients [42, 43], with depression and anxiety being the most frequently reported mental disorders [42, 44], especially in TB patients diagnosed with post-traumatic stress disorder (PTSD) [45]. Besides depression and anxiety, feelings of anger were also reported [13]. Psychiatric co-morbidity may increase the distress of physical illness, prolong recovery time and may also lead to poor treatment compliance [43]. Psychological distress may be caused by social stigmatization followed by social isolation during TB treatment and impacted financial situation [13, 42, 46]. Mental disorder in TB patients was associated with socio-demographic and economic factors and in HIV co-infected TB patients also with stigmatization and perceived health status [15, 42]; a misconception about TB including fear of dying, disease transmission, disease symptoms and treatment leads to lower cure and survival rates as the patient may act according to this misconception [13, 16]. Prevalence rates of depression and mental disorders among TB patients in low income countries ranged between 46 and 80 % [42, 44]. About 20 % of non-adherent TB patients in Pakistan reported depression and anxiety after treatment completion and developed a multi-drug resistance [44]. TB can also have an impact on caregivers. In Malaysia caregivers reported poor mental health and an increased risk of depression [47]. TB may affect spiritual wellbeing. Patients in the USA experienced TB as a wake-up call which changed their lifestyle in a positive way. These patients stopped drinking alcohol, adopted a healthier lifestyle and became more concerned about their health [13].

#### TB impact on social health

Diagnosis and treatment of TB can have an impact on social health including reduced social functioning and an increased financial burden as a result of stigmatization [13]. Social functioning often comprises roles at the workplace, in the community and within the family [3, 13]. The infectious nature of TB can lead to a stigma and disruption of social interaction with others, resulting in social isolation [26]. TB-related stigmatization was often associated with stigma of HIV and AIDS [43, 48]. Many TB patients were unable to work due to the travel distance between a health clinic they get the treatment from and workplace, but also due to the disease induced worsening of their physical condition [3, 13, 16]. This may affect the financial and economic situation of TB patients in terms of limited work capacity or inability to work which could lead to a decrease in income or even a total loss of income [13, 16]. Saving travel expenses to reduce the financial burden maylead to medication non-adherence [3]. Some studies indicated that a better financial empowerment could improve TB-related depression, medication adherence and HRQOL [42, 46]. Often family members needed to get involved as caregivers leading to a loss of income by either through reducing their own work [3] or by providing financial support to the patients [13]. TB induced fatigue may impair the sexual function of the patient [13].

#### South African studies on HRQOL in TB

In this review four studies which evaluated HRQOL in TB patients in South Africa were identified. All of them applied a cross-sectional design and had a strong focus on psychological distress and mental health [22, 42, 43, 45] (Table 3 in the Additional file 1). The evidence shows that TB impacted physical functioning and mental health domains of HRQOL. Mental disorders and psychological distress were present in TB and TB/HIV co-infected patients [42]. Depression was most prevalent (64 %), followed by PTSD (30 %), feeling of helplessness and a lack of social support [43, 45]. It was proposed that the integration of screening, treatment and mental health care services for TB patients [42, 45] would improve the quality of health and health outcomes of TB and HIV co-infected patients in South Africa [22].

#### Adherence to treatment

Medication adherence is a complex, multi-dimensional, dynamic phenomenon comprising patient behavior with regard to the prescribed interval, dose and dosing regimen as well as appropriateness of how the medication is taken [49, 50]. In the absence of drug resistance, TB is a curable disease with a six month treatment of antibiotics [51]. Medication adherence is a key factor for treatment success [52], and might have a greater impact on a population’s health than any improvement in specific medical treatments [53]. Adherence during the intensive treatment phase (first two months) increases the chance for cure in newly diagnosed patients [51]. In contrast, non-adherence leads to spread of free TB bacteria in the community; this may impact the patient by resulting in disability, drug resistance, relapse and risk of death and the community by increased health costs [51, 52, 54, 55]. There is no gold standard on how to measure medication adherence. Conventional ways of adherence control in TB include pill counting and Directly Observed Treatment (DOT); under DOT medication intake is directly observed and thereby it is a surveillance tool to control adherence. A Cochrane systematic review [56] compared DOT with self-supervised TB medication treatment. While DOT is an objective mechanism to control adherence, self-supervised treatment depends on the subjective adherence behavior of the patient. The Cochrane review showed no differences in adherence between the two approaches; further clinic based DOT and community based DOT showed no differences. DOT is a key element of DOTS (Directly Observed Therapy, Short course), a TB management programme from WHO that comprises five elements: government control, detection, medication supply, supervised treatment and monitoring of TB [56, 57]. Although DOTS is commonly practiced, the cure rates in some countries are still low due to poor adherence [57]. A number of studies [14, 40, 41, 43, 47, 48, 50, 54–57] identified qualitatively different factors affecting adherence based on behavioral sciences: therapy-related (adverse events), condition-related (TB symptoms, psychological stress and depression), socio-economic and demography-related (gender, age, food access,education, marital status), health system related (inadequate relationship between health care provider and patient, poor health infrastructure) and patient-related (forgetfulness, drug abuse). Adherence may also be influenced by family pressure, insufficient social support, a fear of disclosure, migration within the country or to neighboring countries. Health beliefs of the patient play a major role in adherence [54, 55] and may impact adherence positively [44, 55, 58–60] or negatively [54, 57]. Material incentives and enablers promote and assist adherence to TB treatment through higher clinic attendance [60, 61]. Material incentives include cash or non-cash vouchers and promoteadherence in form of a positive reward of the patient’s adherence behavior. Enablers assist adherence by directly acting to overcome financial barriers to treatment such as transport vouchers or food; it is unknown whether incentives and enablers will improve adherence in the long-term [61].

#### South African studies on adherence

Four studies assessed adherence to TB medication in South Africa: two cross-sectional studies [62, 63] and two qualitative studies [54, 57] (Table 3 in Additional file 1). These studies indicated that TB patients were more adherent during the first two months of treatment (intensive phase) when symptoms were more present. Patients believed that they could be cured through effective treatment and through a good health alliance with their health care professional [54, 57]. A qualitative study by Naidoo et al. [57] identified a number of themes in a DOTS environment in South Africa which affect adherence behavior. Non-adherence was related to poverty, HIV co-infection, stigmatization, an unsupportive social and work environment, and feelings of helplessness and hopelessness [57]. Although the psycho-social burden in TB was higher than in HIV patients, adherence to both treatments was comparable and the psycho-social burden did not impact adherence [63]. Non-adherence was mainly affected by limited food access, a lack of public transport to clinics, the cost of transport and through social stigmatization [54, 57]. The two cross-sectional studies observed adherence by economic incentives and by patient reported outcomes. One study revealed that material incentives such as monthly vouchers of US$15 given during treatment did improve treatment completion; 35 % of patients receiving no material incentives completed treatment while 43 % of patients receiving vouchers did complete the treatment [62]. However the study was affected by a low fidelity tothe delivery of vouchers in public health clinics and further research into this is required. The other study compared medication adherence between TB and HIV patients by applying the patient-reported Morisky Medication Adherence Scale and by counting missed appointments [63]. On average TB patients missed 1.85 days of treatment and had a good self-reported adherence in this study.

#### The association between HRQOL and adherence during TB

Our systematic review identified one study which addressed the association between well-being and adherence in TB. This study was a meta-analysis of 44 qualitative studies including 8 studies from South Africa about adherence to TB treatment by Munro et al. [5]. The systematic review indicated two underlying mechanisms for the association between well-being and adherence. The first mechanism related to TB patients prematurely stopping their treatment because they felt better. The reason was that patients perceived the improvement in well-being as a cure of TB. On the other hand, the second observed mechanism referred to patients stopping treatment when they experienced no improvement or a worsening in their health status and well-being [5, 53].

#### HRQOL and adherence in longitudinal studies

Ten observational studies with a longitudinal design evaluating HRQOL in new smear-positive TB cases receiving TB treatment were eligible for separate data extraction as shown in Table 1 [10, 17, 19, 26, 28, 29, 39, 64–66]. Three studies took place in India [10, 19, 26], two in Indonesia [17, 66], and one each in Canada, China, Malaysia, the UK and Western Iran [28, 29, 39, 64, 65]. Studies evaluated HRQOL at diagnosis or before treatment, after 4 weeks of treatment, at switch from intensive to continuous phase at two months following treatment initiation, after three months and at the end of treatment after six month. Only one study did not include HRQOL data for the end of treatment time point [39]. Sample size varied from 30 [19] to 1034 [10] with a mean sample size of 206. Seven different HRQOL measures indentified in this review; three were generic (SF-36, WHOQOL-BREF, EQ-5D), three were dimension-specific measures, either for depression (BDI and Center for Epidemiologic Studies Depression Scale), or for anxiety (State-Trait Anxiety Short Form), and one was respiratory-specific (St. George’s Respiratory Questionnaire (SGRQ)). Independent from HRQOL measure and health setting, all studies reported impaired HRQOL before TB treatment and an improvement in HRQOL due to TB treatment. However, it was noted that residual impairment in HRQOL remained in some patients after successful treatment [10, 26, 28, 64]. Variables which were shown to predict lower HRQOL prior to treatment initiation included low socio-economic status and depression [39, 64]. It was also identified from the studies included in the review that HRQOL is most affected in physical and psychological or mental domains [29, 65]. The greatest improvement observed in HRQOL due to treatment was in the physical domain [26, 39].

**Table 1:** Data Extraction of longitudinal studies evaluating HRQOL and adherence in TB

Reference	Study Objective	Study Setting/Sample Size	Population	Comparator Group	HRQOL Measure	Application time pointof HRQOL Measure	Overall Outcome in HRQOL	Outcomes in HRQOL Domains
Aggarwal et al.2013 [10]	To quantify impairment in HRQOL and to evaluate the utility	India N = 1034	Newly diagnosed PTB patients	None	WHOQOL-BREF Hindi version	1st time point: within 2 weeks of initiating intensive phase 2nd time point: within 2 weeks of switching to continuation phase 3rd time point: within 2 weeks of stopping treatment	Impaired HRQOLimproves significantlywith anti-tuberculosistreatment. Residualimpairment is noticedin some patients at theend of treatment	Patients in urban areas and those with higher socioeconomic status (SES) have higher domain scores and better HRQOL. The WHOQOL-BREF physical and psychological domain scores are significantly lower and more affected than other domains.
Atif et al. 2014 [64]	To evaluate the impact of TB treatment on HRQOL	Malaysia n = 216	New smear positive PTB patients; no HIV co-infection	None	SF-36 v2 Tamil, Malay and Mandarin version	1st time point: start of treatment 2nd time point: end of intensive phase 3rd time point: end of treatment	Impaired HRQOL improves significantly with anti-tuberculosis treatment. Scores inthe physical and mental health components were still impaired after end of treatment	Health domains improve between baseline and end of the intensive phase, and end of treatment, except for bodily pain and vitality. At the start of treatment, 67.1 % of patients are at risk of depression, compared to 35 % at end of intensive phase and 23.5 % at end of treatment. Patients aged <45 years and/or non-smokers have a better mean physical component summary (PCS)score. Lower and affected mental health is related to smoking, low income and presence of more than three TB symptoms.
Balgude et al. 2012 [19]	To assess the impact of TB and treatment on HRQOL	India n = 60, (30 patients and 30 controls)	Newly diagnosed smear positive TB patients	Healthy control from the general population	WHOQOL-BREF plus 2 items examined separately	1st time point: baseline 2nd time point: after 2 months 3rd time point: after 4 months	At baseline, HRQOL is significantly affected with physical and psychological domains most affected. All domains improve after 2 and 4 month treatment.	Mean scores of patients’ physical and psychological domains are lower than controls at all 3 time points of assessment. There is significant improvement in the scores at 2 & 4 months of treatment. The mean scores of patients’ environmental and social domains are lower than control at baseline, but improve at 4 months of treatment and are comparable to control
Chamla 2004 [29]	To assess impact of TB and treatment on HRQOL	China n = 205, (102 patients and 103 controls	TB patients	General population without TB	SF-36 Chinese version	1st time point: before treatment 2nd time point: after 2 months 3rd time point: end of treatment.	HRQOL is impaired at baseline with physical scales most affected and improves due to treatment.	Treatment improves all domains; at end of treatment physical functioning, role-emotional, bodily pain, social functioning and general health are not different from control. Physical scales are more commonly affected than mental health scales.
Dhuria et al. 2009 [26]	To assess impact of TB and treatment on HRQOL	India n = 180,(n = 90 patients and 90 controls)	TB patients	General population matching for age, gender and socioeconomic status	WHOQOL-BREF Hindi version	1st time point: baseline 2nd time point: 3 months 3rd time point: end of treatment.	TB patients have an impaired HRQOL with significant improvement in all domains except social domain after treatment.	The highest improvement is in physical domain, followed by psychological domain. The mean score of overall HRQOL and physical domain at completion of treatment is better in females than males. Males score better in psychological, social and environmental domains. After end of treatment HRQOL is still affected in physical domains compared to healthy controls.
Kruijshaar et al. 2010 [39]	To assess the impact of TB and its treatment on patients’ health status	UK n = 61	TB patients	None	SF-36 v2 UK version EQ-5D STAI-6 CES-D	1st time point: diagnosis 2nd time point: 2 months	Impaired HRQOL improves already after 2 month treatment, but is still below the UK norm score	SF-36 v2 scores improve significantly except for physical functioning, general health perceptions and physical summary score. Vitality, mental health and mental health summary scores are comparable to the UK norm. EQ-5D: pain/discomfort andproblems with self-care improvewhile a borderline decrease is seen for mobility, except for self-care. Depression and anxiety improveddue to treatment (CES-D andSTAI-6 scores). 51 % report economic burden due to TB.
Maguire et al. 2009 [66]	To quantify the impact of TB HRQOL	Indonesia n = 115	smear positive PTB patients	None	SGRQ	1st time point: baseline 2nd time point: 2 months 3rd time point: 6 months	Impaired HRQOL improves with treatment at 2 and 6 months	Although HRQOL improves due to treatment 24.6 % of patients still have significant lung function impairment after at end of treatment
Mamani et al. 2014 [65]	To assess the QOL among TB patients	Iran n = 184 (64 patients and 120 controls)	Pulmonary and extrapulmonary TB patients	Healthy control from general population	SF-36 Persian version	1st time point: baseline 2nd time point: 2 months 3rd time point: 6 months	Impaired HRQOL improves due to treatment compared to controls	All domains of SF-36 aresignificantly impaired andimprove after 2 month treatment; improvement betweentwo and six months is not significant. Physical functioningand energy are most affected.
Marra et al. 2008 [28]	To identify areas of HRQOL affected by latent and active TB; treatment impact on HRQOL	Canada n = 206 (104 active TB and 102 latent TB)	Active and latent TB patients (LTBI)	LTBI defined as a positive TST result without radiographic or clinical evidence of active TB	SF-36 v2 BDI	1st time point: baseline 2nd time point: 3 months 3rd time point: 6 months	At baseline HRQOL is more affected in active than latent TB patients. Treatment improves HRQOL in active but not in latent TB. Patients with active TB have still impaired HRQOL after treatment completion compared to US norms.	All domains of SF-36 improve overtreatment in active and latent TBexcept bodily pain inactive andexcept social functioning andvitality in latent TB.BDI showsnoimprovement in LTBIparticipants, but significant improvement for those with active TB.
Ralph et al. 2013 [17]	To investigate morbidity over TB treatment period	Indonesia n = 240, (200 patients and 40 controls)	smear positive TB	Healthy control from the general population	SGRQ Indonesian version	1st time point: baseline 2nd time point: 4 weeks 3rd time point: 8 weeks 4th point time: 24 weeks	Impaired HRQOL improved over treatment time.	After treatment 27 % of TB patients have moderate to severe pulmonary function impairment. HIV -positive status was significantly associated with worse HRQOL
Reference	Study Objective	Study Setting	Population	Comparator Group	Adherence Measure	Application time point of Adherence Measure	Overall Outcome in Adherence	Specific Outcome in Adherence
Chirwa et al. 2013 [51]	To estimate cure rates, and their association with adherence to TB treatment	Malawi n = 524	TB patients	None	Retrospective counting of missing days during treatment	Retrospective review of records	Adherence to TB treatment had a significant effect on cure of TB	Overall, 35.1 % of patients did not fully adhere to TB treatment. Of these, 86.4 % missed < 15 days and 23.4 % missed at least 1 day of treatment Overall, 92.7 % of patients were cured from TB and 33.7 % of these missed at least 1 day of treatment. Patients who missed <15 days and 15 to 29 days of treatment were less likely to be cured compared with those who fully adhered.

Table 1: data extraction from 11 longitudinal studies evaluating HRQOL (10 studies) and adherence (1 study)

*SF-36* short form 36, *EQ-5D* EuroQol 5 dimensions, *SGRQ* St. George’s respiratory questionnaire, *WHOQOL-BREF* World Health Organization Quality of Life Short Form, *BDI* Beck’s depression index, *STAI-6* state-trait anxiety short-form, *CES-D* Center for Epidemiologic Studies Depression Scale

In terms of adherence, there was only one study identified that followed a longitudinal approach [51]. This study assessed medication adherence by counting missing days from patient medical record cards. About 35 % of patients were non-adherent to TB treatment, with the majority missing less than 15 days of treatment. No longitudinal study addressed the association between HRQOL and adherence in TB.

### Discussion

This systematic review addressed the issues relevant to understand the impact of TB on HRQOL and medication adherence from a global perspective and specifically for South Africa using an integrative health approach. TB impacts the physical, emotional, psychological, social and economic dimensions of HRQOL, and residual impairment may be still present after treatment. We identified thirty-six studies evaluating HRQOL in TB of which twenty-one studies took place in WHO’s high-burden TB countries. Atif et al. [67] reported that most HRQOL studies in TB so far applied a cross-sectional design and our systematic review confirms this finding. Thirteen studies across high-burden countries had a cross-sectional design while only eight studies evaluated longitudinal changes (Table 2). To date, very few studies have followed changes in HRQOL longitudinally over time in TB patient populations. No longitudinal study has been conducted in South Africa even though South Africa has the highest prevalence and incidence among the 22 high-burden TB countries worldwide. Our understanding of the long-term impact of TB on HRQOL, covering the time of treatment and after treatment, is limited. There is a need for further research assessing changes in HRQOL longitudinally, specifically in high-burden countries like South Africa. Most studies on medication adherence during TB treatment employed a qualitative approach observing psychometric aspects of adherence. One systematic review of qualitative studies reported an association between HRQOL and adherence TB [5]. We found that similar factors affect HRQOL and adherence including TB therapy, health condition, socio-economic and demographic factors as well as quality of health care services. Studying the association between HRQOLand medication adherence during TB treatment will allow for a better understanding of how treatment effectiveness can be improved and care for TB patients optimised.

**Table 2:** Eligible Studies included in Data Extraction

WHO high-burden country	Total Number of HRQOL studies	Cross-sectional Design	Longitudinal Design
China	2 [29, 69]	1	1
India	6 [10, 12, 19, 26, 33, 70]	1	5
Indonesia	2 [17, 66]		2
Nigeria	2 [14, 46]	2	
Pakistan	1 [55]	1	
Philippines	1 [9]	1	
South Africa	6 [22, 42, 43, 45, 59, 63]	6	
Uganda	1 [11]	1	
Other countries	Total Number of HRQOL studies	Cross-sectional Design	Longitudinal Design
Canada	3 [27, 30, 40]	2	1
Iraq	1 [58]		1
Malaysia	4 [18, 47, 64, 67]	3	1
Taiwan	1 [20]		1
Thailand	1 [21]	1	
UK	1 [39]		1
USA	2 [31, 36]	2	
Western Iran	1 [65]		1
Yemen	1 [23]	1	

Table 2: Representation of eligible studies included in the systematic review and listed according to their WHO high-burden country status

The studies included in this systematic review revealed similar HRQOL outcomes with a number of different measures, but a TB-specific measure is lacking. Although two studies reported developing TB-specific measures, FACIT-TB in Iraq [59] and DR-12 in India [68], neither provides adequate evidence for validity or reliability. Measures applied during longitudinal HRQOL studies were generic (SF-36, WHOQOL-BREF, EQ-5D) and dimension-specific measures, either for depression (BDI and Center for Epidemiologic Studies Depression Scale) or for anxiety (State-Trait Anxiety Short Form), one measure was specific for respiratory diseases (SGRQ). All measures reported similar HRQOL outcomes for TB and confirmed that physical health domains were more affected than mental health domains. All health domains for each measure improved during TB treatment. However, physical impairment was still present after treatment. The measures only capture parts of the health domains relevant to TB or may not be sensitive enough to observe the actual impact of TB on HRQOL. This implies the need for a TB-specific PRO measure which captures all relevant health domains and health aspects of TB including the physical, psychological and social domains. Such a measure will need to consider socio-demographic and cultural differences between patients with TB, taking into account the stigma of HIV and how the social standing of the patient within his or her community is affected. The development of such a specific measure will also allow a deeper understanding of MDR-TB, TB/HIV co-infection and extrapulmonary TB. Future studies should also include the identification of minimal clinically important difference (MCID), i.e. the smallest difference in a domain which patients perceive as an improvement or a worsening; This is unknown for TB patients and will allow understanding meaningful changes in HRQOL.

Current evidence regarding the association between HRQOL and adherence in TB is lacking globally. Future research about the association between HRQOL and adherence in TB specific will help to optimize existing treatment programs, understand the limitations in TB control and targeted interventions and improving the health status of high-burden TB populations. The WHO’s End TB Strategy targets a TB free world by 2035. One of the three strategic pillars to reach this goal focuses on integrated, patient-centred TB care and prevention. HRQOL assessment allows an integrative understanding of TB from a patient perspective. Resulting knowledge supports an understanding of TB-related physical, mental and social needs and addresses diverse barriers. This patient-centred approach may support quality assessment and rationale use of medicines, which fall under the pillars of the End TB Strategy.

This systematic review had several strength and limitations. It combined qualitative and quantitative research on HRQOL and adherence and this mixed method may be viewed as strength as it ensures capturing all relevant information on the topic. The methodological quality of the eligible studies varied and might have an effect on the reported outcomes. We only applied the STROBE Statement for quality reporting to longitudinal studies; we observed a moderate reporting quality with a median of 7 out of 16 points, indicating that the quality of the articles included in this review were fairly poor. This might affect the reported information. Most studies applied different PRO measures at differing time points during treatment at different study sites, making any general conclusions regarding the impact of TB on HRQOL difficult. Studies had an observational nature rather than a controlled trial design as mostly applied in systematic reviews; however, observational studies might be more reliable as they observe patients in real life rather than under controlled ideal conditions. Publication bias may be present in this systematic review although our findings were consistent across different settings; we selected peer-reviewed literature from three different databases and included grey literature to control for publication bias. We did not include unpublished information and studies published in a different language than English.

## Conclusions

The relevance and importance of HRQOL assessments is growing and HRQOL has become an important tool for the understanding of health outcomes adopting a patient-centred approach to care and treatment. Further research is required in a country-specific context to contribute to efficient decision making with regard to TB related strategies; product approval, pricing and reimbursement as well as health policy making; A number of new anti-TB drugs, vaccines and diagnostics have recently achieved marketing approval or are in late clinical development.Assessment of longitudinal changes in HRQOL and its association to medication adherence in TB in a high TB burden country such as South Africa are not yet available. Such data would support the identification of sustainable health innovations in TB by providing information on benefits and gaps in current treatment strategies tailored for a specific patient population. This will be useful to determine the value of new products and to perform appropriate cost-effectiveness analyses to optimize the allocation of societal resources specifically for South Africa. This is of high relevance as a number of new TB drugs, vaccines and diagnostics have recently achieved marketing approval or are in late clinical development. A longitudinal study assessing HRQOL and medication adherence in active TBin South Africa applying valid and reliable measures that capture all relevant aspects of TB would be necessary to assess the achievement of the WHO’s End TB Strategy pillars.

## Additional file

Additional file 1: I. Search strategy for identification and selection of relevant studies. II. Quality of reporting check list for longitudinal studies. III. Studies on HRQOL and medication adherence performed in South Africa. (DOCX 682 kb)
