# Supplementary material for: Health-related quality of life and its association with medication adherence in active pulmonary tuberculosis– a systematic review of global literature with focus on South Africa
Source: Health Qual Life Outcomes. 2016 Mar 11;14:42. doi: 10.1186/s12955-016-0442-6 (PMC4788905; doi:10.1186/s12955-016-0442-6)
Supplement: Additional file 1: — I. Search strategy for identification and selection of relevant studies. II. Quality of reporting check list for longitudinal studies. III. Studies on HRQOL and medication adherence performed in South Africa. (DOCX 682 kb) [file 12955_2016_442_MOESM1_ESM.docx]

# Additional File 1

## Search strategy for identification and selection of relevant studies

A systematic and comprehensive literature search was performed in PubMed, EMBASE and PsychINFO, with the last search conducted on 22 February 2015. Search terms applied included *tuberculosis, health related quality of life, HRQOL, quality of life, South Africa, patient-reported outcomes, outcome assessment, life quality, well-being, adherence, non-adherence, and compliance.* All search terms were combined differently and applied to each database, as shown in table 1:

**Table 1: Combination of search terms applied to Pubmed, EMBASE and PsychINFO**

| Search Term combinations applied to Pubmed, EMBASE and PsychINFO |
| --- |
| Tuberculosis AND health related quality of life |
| Tuberculosis AND HRQOL |
| South Africa AND health related quality of life |
| Tuberculosis AND South Africa AND health related quality of life |
| Tuberculosis AND patient-reported outcomes |
| Patient-reported outcomes AND South Africa |
| Tuberculosis AND patient-reported outcomes AND South Africa |
| Tuberculosis AND well-being AND outcome assessment AND life quality |
| Pulmonary Tuberculosis AND adherence AND South Africa |
| Tuberculosis AND adherence AND health-related quality of life |
| Tuberculosis AND non-adherence AND quality of life |
| Non-adherence AND health-related quality of life |
| Non-adherence AND health-related quality of life AND tuberculosis |
| Non-adherence AND health-related quality of life AND South Africa |
| Tuberculosis AND compliance AND quality of life |
| Tuberculosis AND compliance AND quality of life AND South Africa |

**Table 1: Search terms were combined differently 16 times and applied to the databases PubMed, EMBASE and PsychINFO.**

Each search term combination was applied to the database separately and resulted in “initial hits”. Initial hits were then screened by title and abstract with regard to search terms and selected articles resulted in “Hits by abstract and title”. Articles were excluded if they were not related to the pre-defined search terms or were published in a language other than English. From the selected articles, duplicates were removed and resulted in “Hits after removal of duplicates”.

The following figures 1-3 present each search term combination with its related initial hits and number of articles after review by title and abstract and removal of duplicates, for each database.

**Figure 1: Search Strategy in Pubmed**

**
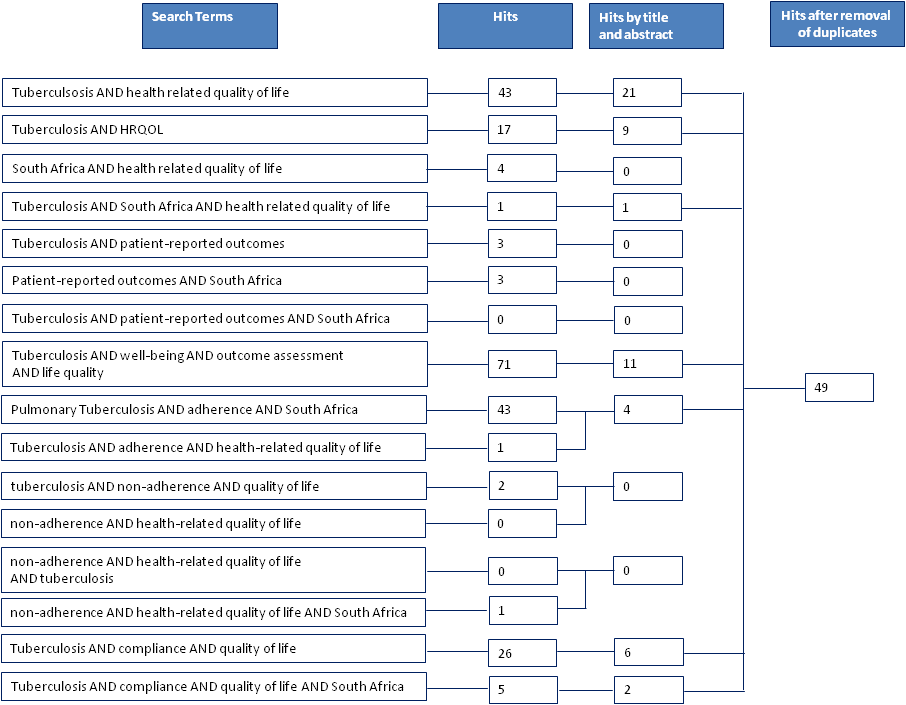
**

**Figure 2: Search Strategy in EMBASE**


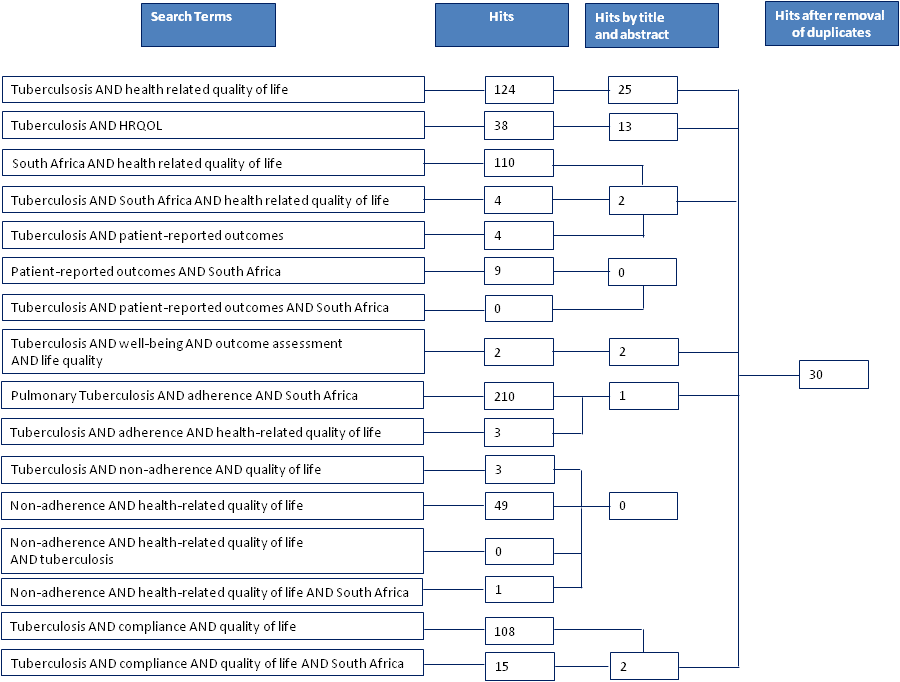


**Figure 3:** **Search Strategy in PsychINFO**


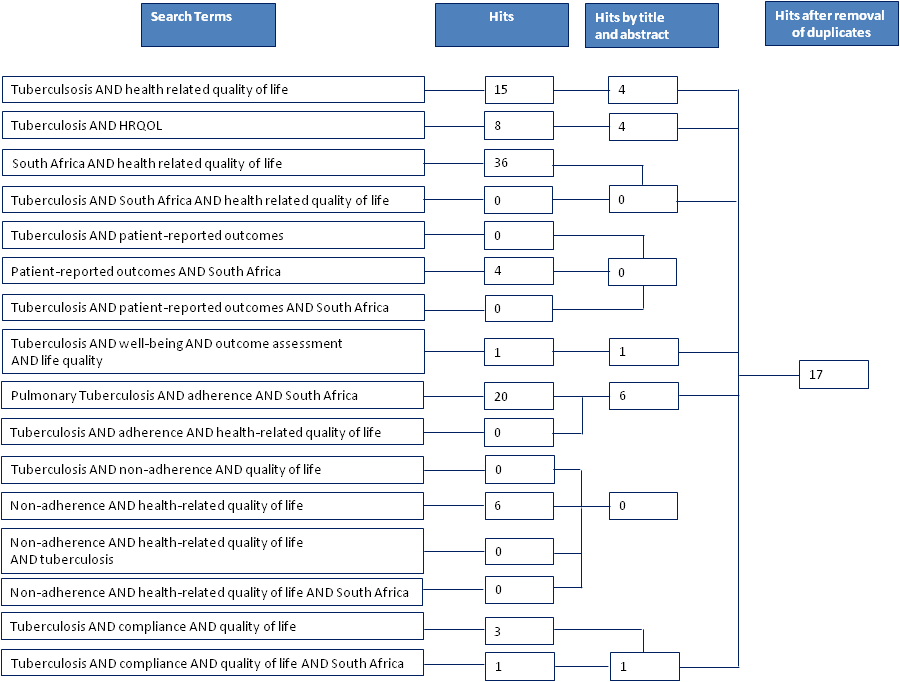


**Figure 1-3: Search terms were applied to the databases PubMed, EMBASE and PsychINFO and hits were screened by title and abstract and duplicates were removed.**

Our literature search in PubMed, EMBASE and PsychINFO yielded 988 initial hits; after screening by title and abstract and after removal of duplicates, 61 articles remained. Addition of three WHO reports, two guidelines from Department of Health South Africa and one article identified by hand search of citations resulted in 67 eligible articles for this systematic review. One article was excluded, yielding 66 eligible full-text articles (Figure 4). A detailed description of the literature search is available in the Appendix. The 66 articles comprised 22 cross-sectional studies, 17 longitudinal studies, 7 (systematic) reviews, 8 qualitative studies and 12 articles including editorials, comments and letters. Nine studies were performed in South Africa (four HRQOL and five medication adherence studies; Table 4). All final 66 articles underwent extraction of information about HRQOL and adherence in TB. The 17 identified longitudinal studies were potentially eligible for separate data extraction; 11 of them actually met the eligibility criteria for separate data extraction, while 6 studies were excluded as eligibility criteria were not met (Table 3). Application of the STROBE quality of reporting checklist to the 11 longitudinal studies resulted in a median score of 7 for HRQOL studies, with scores ranging from 5 to 11 out of 16 (the greater the score the higher the quality of reporting). The adherence study had a score of 10 (Table 3).

**Figure 4: Flow diagram of literature search**


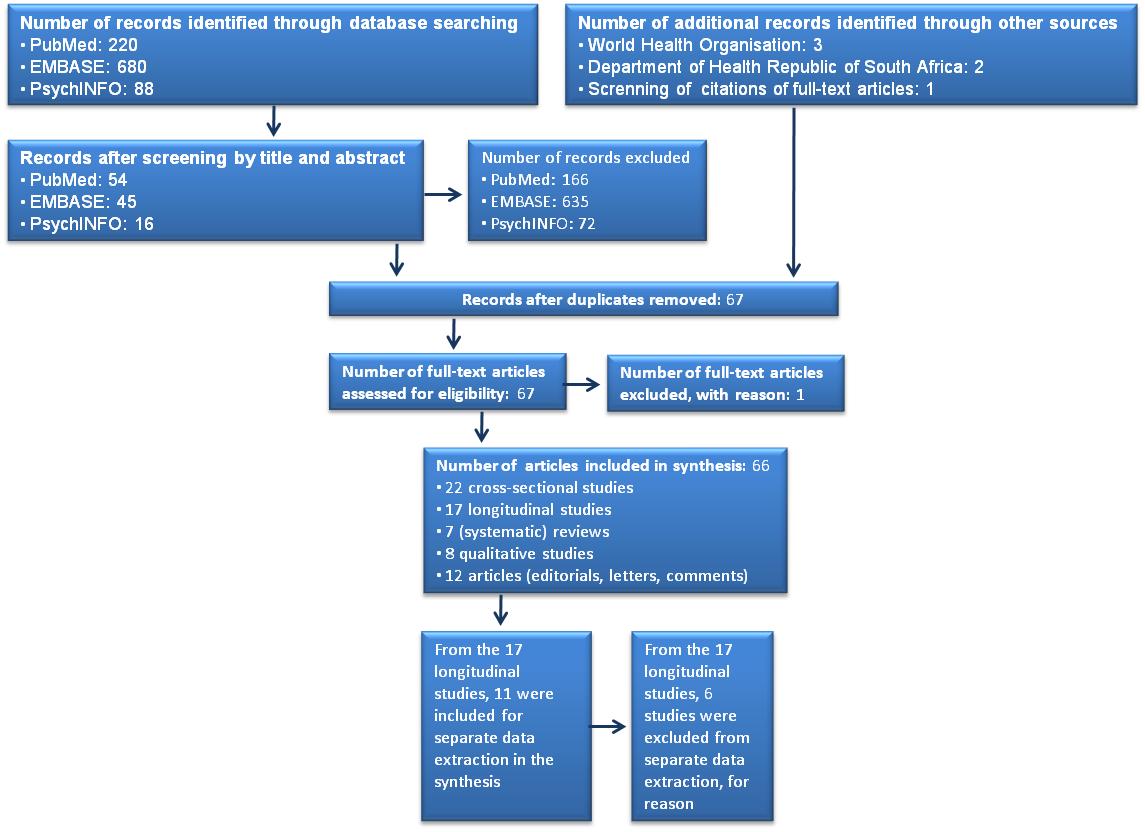


**Figure 4: Literature search process in the databases PubMed, EMBASE and PsychINFO; additional articles were obtained from World Health Organization and Department of Health Republic of South Africa.**

All final 66 articles were uploaded to EndNote X7 version 3.1. Table 2 presents all 66 identified articles included in this systematic review.

**Table 2: Articles finally included in the systematic review**


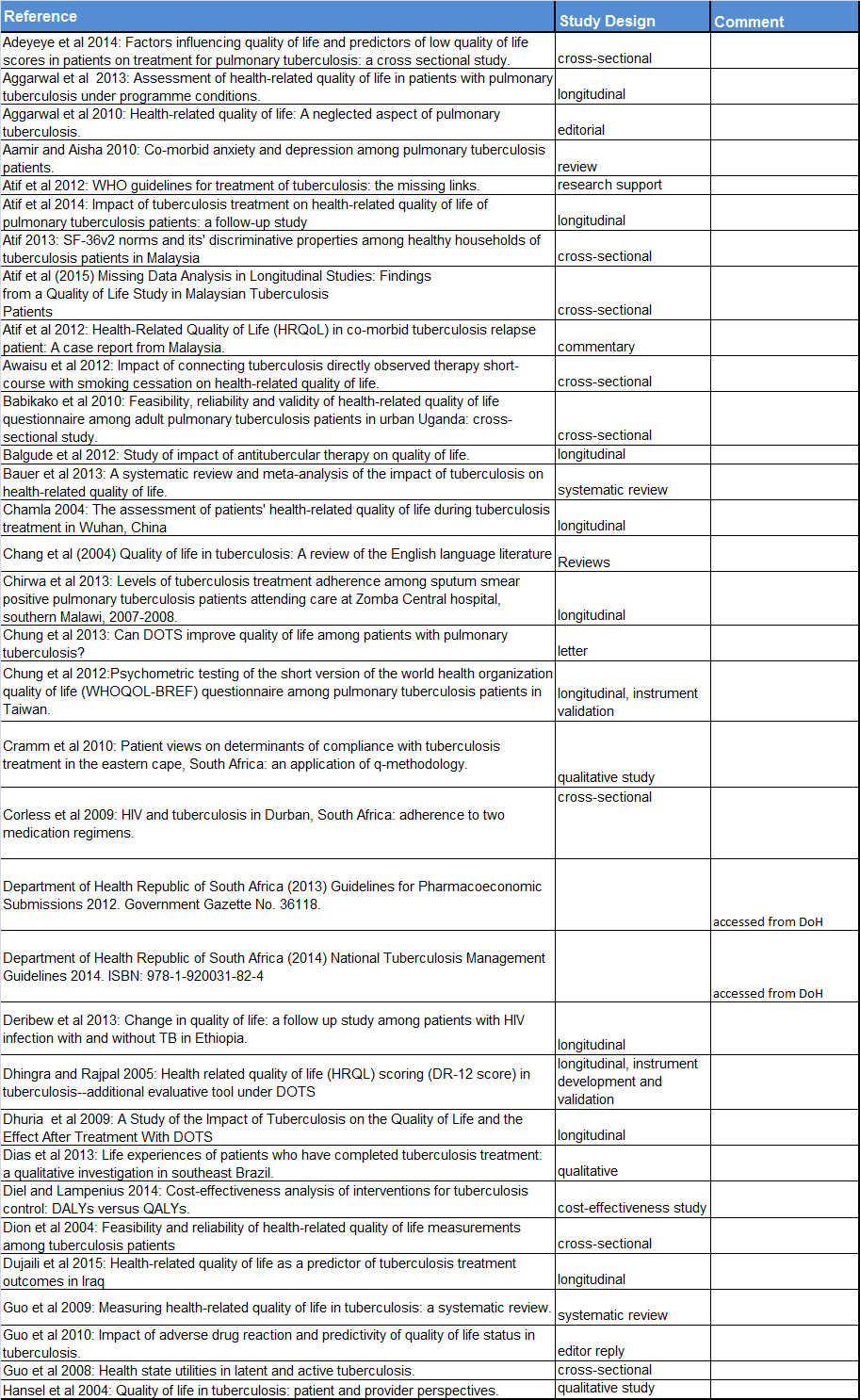


**Articles finally included in the systematic review (cont.)**

**Table 2: Final eligible articles included in the systematic review.**

## Quality of reporting check list for longitudinal studies

**Table 3: Quality of reporting of longitudinal studies in HRQOL and medication adherence in PTB according to STROBE Statement and Bauer et al (2013)**


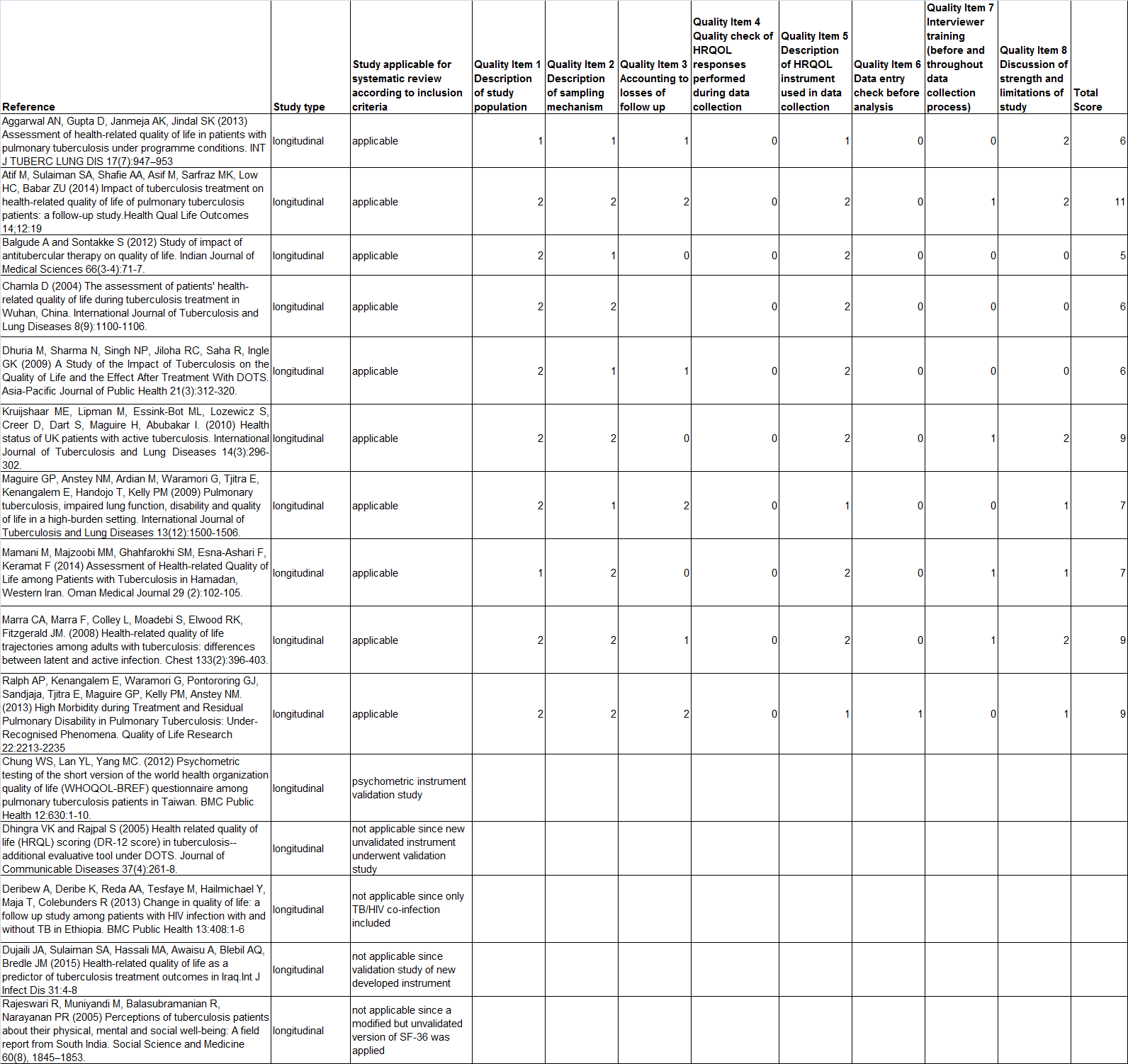


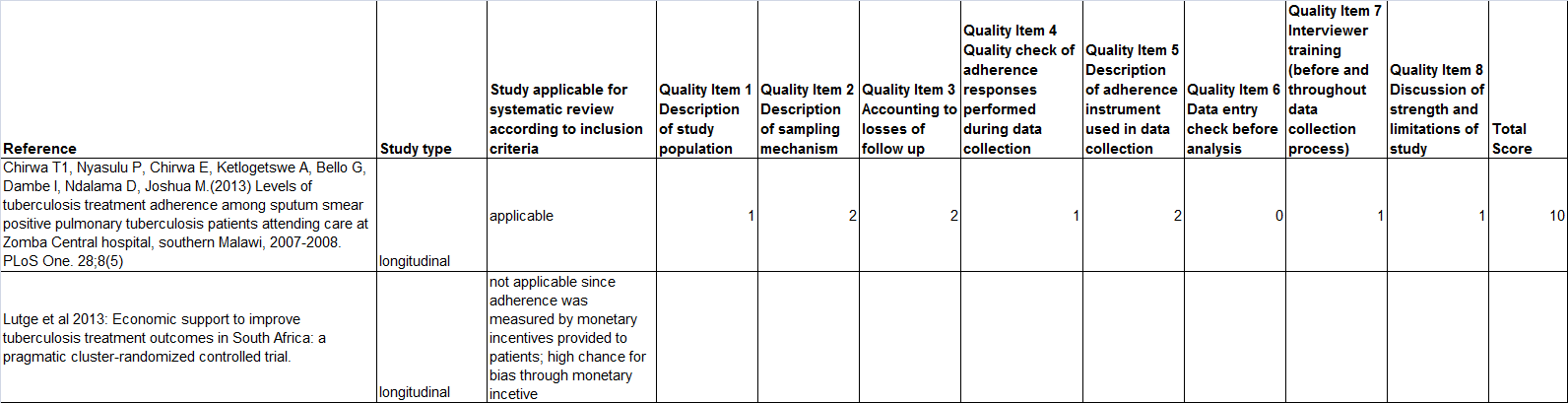


**Table 3: Quality of reporting check of longitudinal studies evaluating HRQOL and adherence, which were used for data extraction; reasons for excluding studies from quality reporting check and data extraction are mentioned.**

## Studies on HRQOL and medication adherence performed in South Africa

**Table 4: Studies on HRQOL and medication adherence performed in South Africa**


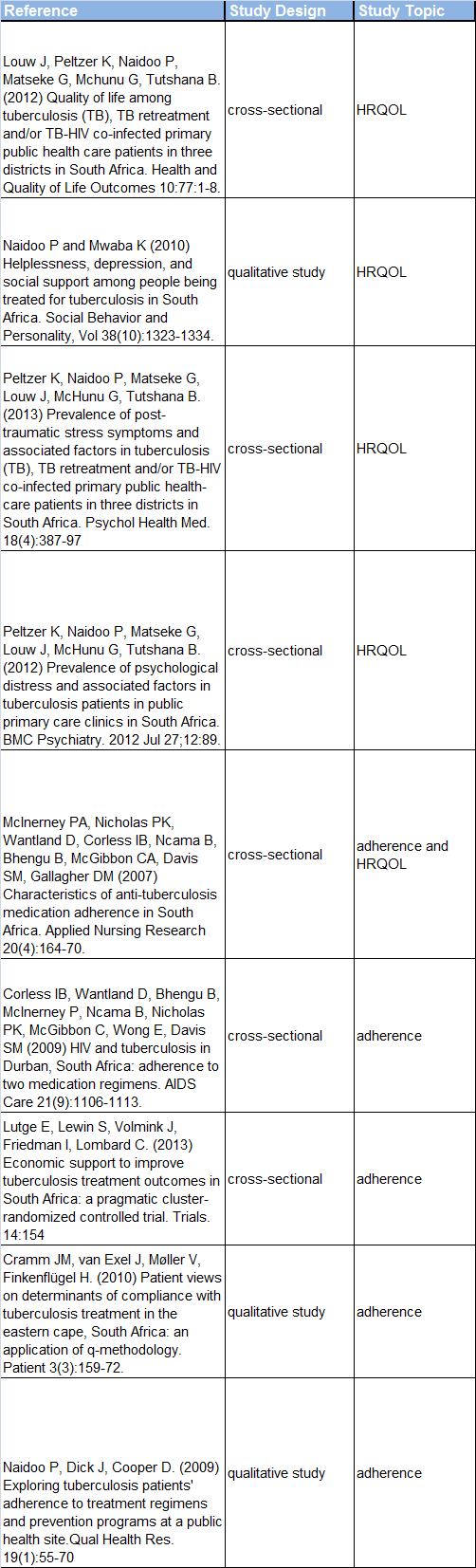


**Table 4: from all eligible articles included in the systematic review nine articles reported research performed on HRQOL and adherence in South Africa.**
